# Increasing Virulence in Leprosy Indicated by Global *Mycobacterium* spp.

**DOI:** 10.3201/eid2401.171785

**Published:** 2018-01

**Authors:** William Levis, Tina Rendini, Frank Martiniuk

**Affiliations:** Bellevue Hospital Center, New York, New York, USA (W. Levis, T. Rendini);; JME Group, Roseland, New Jersey, USA (F. Martiniuk)

**Keywords:** Mycobacterium lepromatosis, bacteria, M. leprae, Lucio’s phenomenon, leprosy, leprosy bonita, autochthonous, armadillo, zoonosis, multidrug therapy, virulence, United States, Singapore, China, India

**To the Editor:** The November 2017 issue of *Emerging Infectious Diseases* had 3 articles about leprosy, including these topics: a United States–born patient who tested positive for *Mycobacterium* *lepromatosis* (*1*); a lethal case of *Mycobacterium leprae* manifested as Lucio’s phenomenon in Peru (*2*); and pointing out that leprosy is an emerging disease in the eastern United States, including autochthonous cases without exposure to armadillos (*3*), which were previously shown to be a zoonotic source of transmission in the United States (*4*). Not only is leprosy not disappearing in the United States and globally, but the signs are pointing to a more virulent mycobacterial infection that is likely to be a microbial adaptation to the global use of multidrug therapy, as previously reported (*5*). 

Lucio’s phenomenon is fortunately rare; there is no proven effective therapy for this type 3 reaction in leprosy patients. Historically, Lucio’s phenomenon was confined to Mexico, mostly in cases of diffuse lepromatous leprosy, also referred to as “Leprosy bonita.” In recent years, it has been discovered elsewhere, including the first known case in India in 2001 (*6*). Two additional cases of lethal Lucio’s leprosy were reported in 2 immigrants from Singapore to the United States, who were shown to have *M*. *lepromatosis* and *M.* *leprae* (*7*). The report of Levis et al. is likely confirmed by the recent discovery of *M*. *lepromatosis* and Lucio’s outside of Mexico (*5*).

In summary, leprosy is an emerging infection in the United States, including autochthonous cases in the eastern United States. The reports in the November issue of *Emerging Infectious Diseases* of autochthonous *M*. *lepromatosis* and a lethal case of Lucio’s phenomenon outside Mexico are ominous signs of a more virulent form of emerging leprosy.
